# Isolation and characterization of cellulase producing bacteria from forest, cow dung, Dashen brewery and agro-industrial waste

**DOI:** 10.1371/journal.pone.0301607

**Published:** 2024-04-10

**Authors:** Mulugeta Samuel Demissie, Negash Hailu Legesse, Aderajew Adgo Tesema

**Affiliations:** 1 Department of Biology, College of Natural and Computational Science, Debre Berhan University, Debre Berhan, Ethiopia; 2 Department of Biotechnology, College of Natural and Computational Science, Woldia University, Weldiya, Ethiopia; University of Kalyani, INDIA

## Abstract

The continuous accumulation of waste, particularly from industries, often ends up in landfills. However, this waste can be transformed into a valuable resource through innovative methods. This process not only reduces environmental pollution but also generates additional useful products. This study aims to screen novel high-efficiency cellulose-degrading bacteria from cow dung, forest soil, brewery waste, and agro-industrial waste in the Debre Berhan area for the treatment of cellulose-rich agricultural waste. The serial dilution and pour plate method was used to screen for cellulolytic bacteria and further characterized using morphological and biochemical methods. From eleven isolates cow dung 1 (CD1), cow dung 6 (CD6) and cow dung (CD3) which produced the largest cellulolytic index (3.1, 2.9 and 2.87) were selected. Samples from forest soil, and spent grain didn’t form a zone of clearance, and effluent treatment and industrial waste (IW9) shows the smallest cellulolytic index. Three potential isolates were then tested for cellulolytic activity, with cow dung 1 (CD1) displaying promising cellulase activity. These bacterial isolates were then identified as Bacillus species, which were isolated from cow dung 1 (CD1) with maximum cellulase production. Cow dung waste is a rich source of cellulase-producing bacteria, which can be valuable and innovative enzymes for converting lignocellulosic waste.

## Introduction

Urbanization in Ethiopia has brought a major problem for the disposal of agricultural residue. Every year, a significant amount of this residue is generated, mainly composed of cellulose, making up 80% of the waste materials in most towns [1,2]. Cellulose is a polymer made up of linear D-glucose molecules connected by 1,4-glycosidic linkages and is a crucial structural component of plant cell walls. Its partially crystalline structure provides extra strength and makes it less susceptible to enzymatic breakdown than other polysaccharides in the cell wall [3]. Composting is the main method used for recycling cellulose waste, but refractory cellulose poses a challenge to efficiently decomposing agricultural waste [4]. It comprises 35–50% of plant dry weight and is also associated with other lignocellulosic biomass like hemicellulose and lignin that make 20–35% and 5–30% of the plant dry weight, respectively. The enzyme cellulase catalyzes the breakdown of cellulose into sugars [5].

Cellulases are widely used in industries like laundry, food, textiles, pulp and paper, and agriculture, making them a highly sought-after enzyme [6]. They currently make up 8% of the global industrial enzyme market and are expected to see a 100% increase in demand by 2014 [7]. The growing concern about the scarcity of remaining energy and the generation of greenhouse gases as a result of incomplete combustion of fossil fuels, which causes air pollution, has brought attention to the use of cellulases for enzyme-driven breakdown of lignocellulosic waste [8,9].

Scholars have been emphasizing various bacteria that produce cellulases since they have a faster growth rate and are more resistant to unfavorable conditions than fungi and have a significant prospect for cellulase production [10]. Bacterial isolates such as *Pseudomonas fluorescens*, *Bacillus subtilis*, *E*. *coli*, and *Serratia marscens* can produce a cellulase enzyme (0.2–1 U/mL) that fully breaks down cellulose to glucose for growth and development purposes [11]. Carboxymethylcellulose (CMC) was identified as the most effective medium for screening and producing cellulase-producing bacterial isolates [12]. As a result, cellulase’s low manufacturing cost and high enzyme efficiency boost its commercial significance [13]. Waste materials generated by industrial and agricultural activities include fruit and vegetable peels, sugarcane bagasse, sawdust, and agro-residues. These components have a greater cellulose percentage, making them cheaper sources of substrate for cellulase synthesis; nevertheless, the presence of lignin inhibits the pace of disintegration. Therefore, to break down lignocellulosic resources cellulolytic bacteria must be used [14].

Bacteria are becoming increasingly favored for the purification and production of various enzymes due to their faster growth rate, numerous enzyme complexes, and ability to withstand a wide range of external difficulties [15,16]. Furthermore, they have been demonstrated to use a wide range of substrates, including solid organic waste leftovers from agriculture, forestry, and mills, to provide waste management benefits as well as economic enzyme production [17]. Bacteria are an intriguing microbial group for faster cellulase production due to their faster growth rate than fungi, ease of handling, and ability to react to a wide range of genetic changes [18,19]. This study aims to screen for novel high-efficiency cellulose-degrading bacteria from cow dung, forest soil, brewery waste, and agro-industrial waste around the Debre Berhan area for the decomposition of cellulose-rich agricultural waste. The isolates are initially screened using the carboxymethyl cellulose (CMC) culture method using Congo red staining. high-efficiency cellulose-degrading bacteria are identified through morphology and biochemical testing. Growing conditions will be optimized for maximum cellulase production. This research will lead to better commercial use of the bacteria for cellulose bioconversion and safe management of cellulose-rich agricultural waste.

## Materials and methods

### Description of the study area

The current study was done from September 2022 to June 2023. The research location was chosen using the purposive sampling method in Debre Berhan, Ethiopia. Debre Berhan is found in the North Shewa Administration Zone, Amhara National Regional State (ANRS), Ethiopia, at 9°40’N and 39°30’E. The town has a total population of 110,000 people and an area of 18,081.95 acres. It is made up of 5 sub-cities, based on Debre Berhan’s new administration (personal correspondence with North Shewa Administrative Zone Office, April 21, 2022). Recently the city of Debre Berhan has established itself as an alluring agro-processing investment destination, industrial zone and a mass of cellulose-rich wastes disposed to landfill so Debre Berhan City can be a good area selected for this study.

### Sample collection and preparation

Samples were collected from spent grain (SG) and hot trub at the Effluent Treatment Plant (ETP) which remains after the mashing and lautering process were obtained from the Dashen brewery industry Debre Berhan. 500g spent grain and hot trub samples were collected using a sterilized polythene bag, sealed and labeled. Forest soil, Industrial waste, and cow dung samples were collected randomly from where cows spend their days and industrial waste at municipal waste dumping areas were collected using sterile Schott bottles around Debre Berhan, Ethiopia, and samples were stored at 4°C for further use.

### Isolation of bacteria

Ten grams of forest soil, animal dung, or ten milliliters of effluent sample, brewery spent grain, and industrial waste were combined separately with 90 ml of sterile distilled water and mixed for 20 minutes by agitating. Then, further serial dilution was done by adding 1mL of the aliquot into 9 mL of sterile distilled water to make an appropriate dilution (10^−5^–10^−6^). Finally, 100μl of the aliquot from appropriate dilution was spread on Carboxy Methyl Cellulose (CMC) agar media (0.2% K₂HPO₄, 1% CMC % agar, 0.03% MgSO₄.7H₂O and 0.25% (NH₄)₂SO₄ at pH 7) and incubated at 37± 2°C for 48 hrs. The bacteria were sub-cultured, purified, and preserved at 4°C for further screening of cellulose-degrading bacteria [20,21].

### Screening of cellulase-producing bacteria

For primary screening isolated bacteria grown on 1% carboxymethylcellulose media [22] and incubated at 30 ± 2°C for 15 minutes, the Petri dishes were flooded with Congo red solution (0.1%w/v) [23]. The reagent solution was discarded and the plates were washed with 1M NaCl solution for 15–20 minutes. A clear zone around the colony indicates the presence of cellulase enzyme [24]. Bacteria isolates capable of decomposing CMC were identified by the emergence of a clear zone surrounding the colony after testing with Congo red. The diameter was measured with a caliper to calculate the Cellulolytic index (CI). The higher the cellulolytic index, the more cellulase enzyme is produced by bacteria. Cellulase enzyme activities were graded based on the cellulolytic index value, with a low category if the CI < 1, medium if the CI value = 1 to 2, and high if the CI > 2. Further study was conducted using an isolate with a high cellulolytic index value. The following formula was used to calculate the Cellulolytic index [25].


$$Cellulolytic\;index=\frac{Diameter\;of\;zone-Diameter\;of\;colony}{Diameter\;of\;bacterial\;colony}$$

### Bacterial isolate identification and characterization

#### Characterization of isolates using macro and microscopical methods

The cellulase-producing isolates were characterized morphologically and biochemically using Bergey’s manual of determinative bacteriology. The bacterial cultures were freshly grown on nutrient agar media using the streaking method to observe colony shape [26]. A bacterial smear was made and left to air dry before being heat-fixed by rapidly passing the slide over the flame of a Bunsen burner for gram staining on a clean glass slide. Once the bacteria were stained the smear was air-dried and examined under a microscope. Morphology of the colony such as texture, size, and color were recorded also gram staining and motility tests were performed [10].

#### Biochemical tests

In this experiment, different biochemical tests were carried out following [27] protocol, citrate utilization test, catalase test, carbohydrate (Glucose, Lactose, Sucrose) fermentation test and triple sugar iron tests were performed with minor modification [28].

### Cellulose-producing bacteria screening

#### Preparation of the inoculum

A loop of culture was moved to the LB medium for inoculum preparation, and the starting medium was transferred to the production medium [29]. The basal medium contains the following ingredients: 0.2% KH_2_PO_4_, 0.7% K_2_HPO_4_, 0.1% yeast extract, 0.01% MgSO_4_, 0.05% sodium citrate, and 1% carboxymethyl cellulose (CMC) as a carbon source for cellulase synthesis. The pH of the media was then fixed to 7. Autoclaved production medium (50 ml) inoculated with 1 ml of bacterial isolate inoculum. The medium was incubated for 72 hours in a rotary shaker at 200 rpm and 37°C. After 72 hours, 10 ml of culture was centrifuged for 15 minutes at 1000 rpm. The cell-free extract was then subjected to an enzyme assay [20].

#### Enzyme assay

The isolate was cultured in a basal medium with a maximum zone of hydrolysis and incubated overnight at 37°C. The culture was centrifuged and the clear supernatant was used as a crude enzyme source. The level of reducing sugars generated during hydrolysis was used to determine the activity of cellulose enzyme using the DNS (3,5-dinitro salicylic acid) technique. As a substrate, 1% CMC was used in 1N citrate buffer (pH 5.0). 1 ml CMC solution was mixed with 100μl crude enzymes and 1 ml citrate buffer (pH 5.0). The combination was incubated at 45°C for 30 minutes before DNS was added to the solution and the reaction was stopped [30]. Optical density was read at 540 nm once the sample boiled and cooled at room temperature. The enzyme assay was carried out in triplicate in all cases [31]. The cellulase activity was calculated using the following formula.


$$Activity\;(U/mL)=\frac{reducing\;sugar(mg)*1000}{Mm\;reducing\;sugar*30\min*0.1\;mL}$$

The reducing sugar content is the amount of reducing sugar which is formed (mg) and determined by the DNS method; Mm reducing sugar is simplified by using the molar mass of glucose (180 g/mol), incubation time is the hydrolysis period of cellulose (30 min), and sample volume is the amount of crude extract analyzed (0.1 mL). Cellulase activity was measured in U/mL. 1 unit is defined as the amount of enzyme required to liberate 1 μmol of reducing sugar per minute, under the assay condition [29].

#### Enzyme optimization

Different tests were done on selected bacteria isolates using CMC broth media for cellulase production. Effects of factors on the activity of the enzyme were determined by measuring the cellulase activity at different pH values (3, 4, 5, 6, 7, 8, and 9), temperature (25, 30, 35, 40, 45°C), incubation period (24, 36, 48, 72 and 96h) at 37°C. Substrate concentration (0.5%, 1%, 1.5%, 2%, and 2.5%) to test the highest cellulase production. For maximum cellulase production, different types of carbon sources (glucose, sucrose, and starch) and substrates (CMC) were used [32]. To examine the impact on enzyme synthesis, several forms of nitrogen supplies (yeast extract, beef extract, and peptone) were employed [12].

### Data analysis

Colony size and cellulolytic index parameters and descriptive analysis were performed for morphological and biochemical characterization. One-way ANOVA was performed using IBM SPSS Statistics (version 26) software. A Post Hoc test was applied to determine the significance of differences between different factors. All experiments were performed in triplicate, differences were considered significant at *p* < 0.05.

## Results

### Isolation and screening of cellulase-producing bacteria

Based on colony morphology, 34 distinct colonies were isolated from the nutrient agar from these 34 colonies ten were from industrial waste, eight were from Dashen brewery waste, five were from forest waste, five were from spent grain waste and six were from Cow dung (Table 1). They were selected and streaked on starch agar plates. From these, using the Congo red solution, 11(32%) isolates were observed to give a zone of clearance around their colonies. About 23 (68%) of the isolates showed no cellulase activity (Table 1).

**Table 1:** Clear zone formation for 34 distinct colonies.

Isolate	Clear zone	Isolate	Clear zone
IW1	-	ET8	-
IW2	+	FS1	-
IW3	-	FS2	-
IW4	-	FS3	-
IW5	-	FS4	-
IW6	-	FS5	-
IW7	-	SP1	-
IW8	+	SP2	-
IW9	+	SP3	-
IW10	-	SP4	-
ET1	+	SP5	-
ET2	-	CD1	+
ET3	+	CD2	+
ET4	-	CD3	+
ET5	+	CD4	-
ET6	-	CD5	+
ET7	-	CD6	+

CD = Cow dung, ET = Effluent treatment, FS = Forest soil, IW = Industrial waste, SG = Spent grain, + = Colonies which form clear zone,— = Colonies with no clear zone formation.

### Screening of the cellulase-producing bacteria

**Fig 1:**
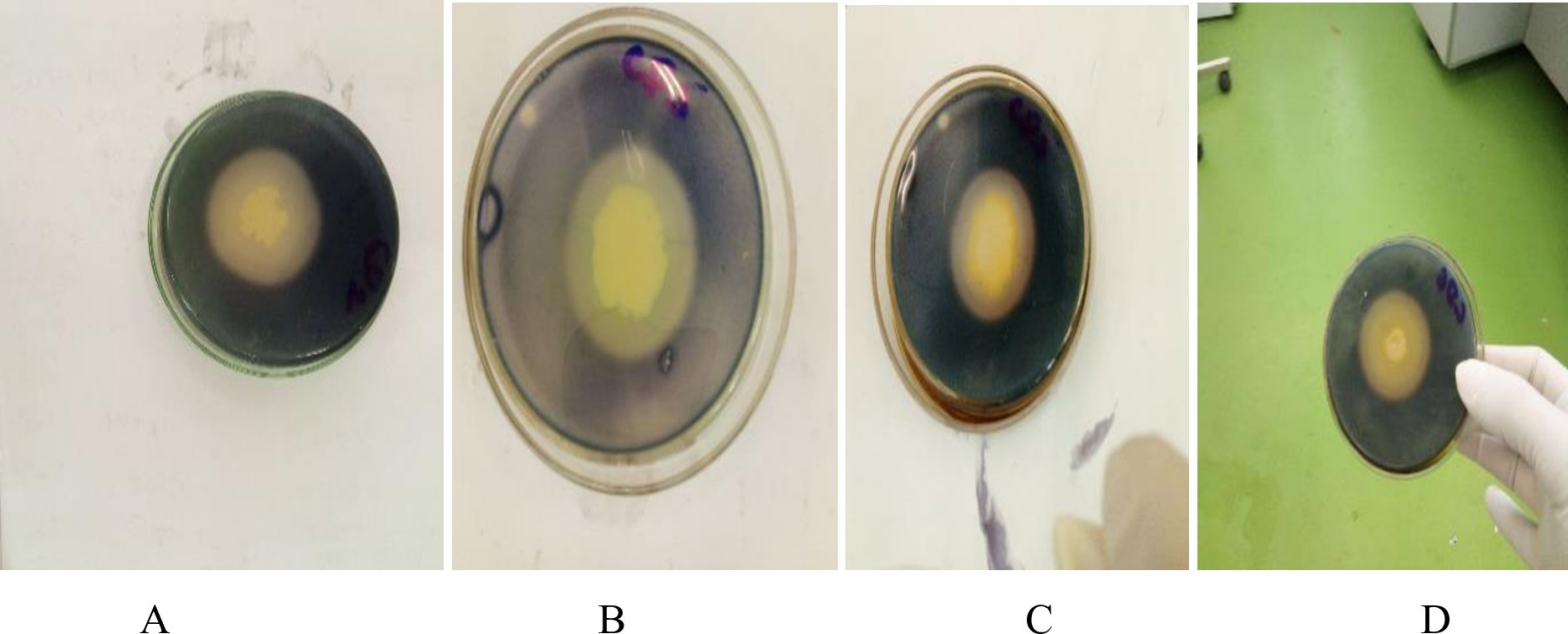
Zone of hydrolysis of cellulose by four selected isolates. A: Cow dung isolate 1(CD1), B: Cow dung isolate 2 (CD2), C: Cow dung isolate 3(CD3), D: Cow dung isolate 6(CD6).

Eleven positive isolates were selected for further investigation. The selection of potent bacteria was done by comparing the isolates with each other in terms of the diameter of the clear zone of hydrolysis (Table 2). The results showed that the isolates with a higher clear zone of hydrolysis also assumed higher cellulase activities (Fig 1).

**Table 2:** The mean value ± standard deviation for the clear zone, Colony size, and cellulolytic index formed by selected bacterial isolates.

Isolate	Diameter of clear zone (mm)	Colony size (mm)	Cellulolytic index (mm)
CD1	4.50±0.1	1.45±0.01	3.10±0.1
CD2	4.45±0.01	2.11±0.04	2.11±0.01
CD3	4.3±0.1	1.55±0.01	2.87±0.02
CD5	3.97±0.2	2.35±0.03	1.69±0.01
CD6	4.35±0.01	1.49±0.01	2.91±0.01
ET1	2.10±0.1	1.35±0.02	1.56±0.01
ET3	2.45±0.02	1.35±0.03	1.82±0.01
ET5	2.35±0.03	1.20±0.02	1.96±0.01
IW2	2.2±0.17	1.35±0.04	1.63±0.01
IW8	2.25±0.02	1.25±0.01	1.80±0.01
IW9	2.20±0.17	2.00±0.2	1.10±0.1

CD = Cow dung, ET = Effluent treatment, IW = Industrial waste.

As can be seen from Table 2, the highest clear zone of hydrolysis (4.5mm) was obtained from CD1, followed by CD2 (4.45mm) while the lowest clear zone of hydrolysis (2.1mm) was obtained from ET1, followed by IW9 (2.2mm) and IW2 (2.2mm). The largest colony size (2.35mm) was obtained from CD5, followed by CD2(2.11mm) while the smallest (1.2mm) was obtained from ET5, followed by IW8(1.25mm) and IW2(1.35mm). The highest cellulolytic index (3.1) was obtained from CD1, followed by CD6 (2.91) while the lowest cellulolytic index (1.56) was obtained from ET1, followed by IW9 (1.1). The diameter of the average clear zone and colony diameter which was produced within different isolates showed significant differences (*P*≤0.05).

### Identification and characterization of the selected bacterial isolates

The morphological characteristics of the bacterial isolates are summarized in (Table 3). Among 11 cellulose-producing isolates, eight were white, two were watery and one was yellowish. From 11 cellulose-producing isolates, four were irregular, two were mucoid, two were raised and one was oval. In colony size from 11 cellulose-producing isolates five were medium, three were large and three were small (Table 3).

**Table 3:** Morphological characterization of bacterial isolates.

Isolate	Colony characteristics		
	Color	Texture	Size
CD1	White	Mucoid	Medium
CD2	White	Mucoid	Medium
CD3	Watery	Raised	Small
CD5	White	Irregular	Large
CD6	White	Irregular	Medium
ET1	White	Irregular	Large
ET3	Watery	Raised	Small
ET5	Yellowish	Circular	Medium
IW2	White	Irregular	Medium
IW8	White	Circular	Small
IW9	White	Ovale	Large

CD = Cow dung, ET = Effluent treatment, IW = Industrial waste.

For biochemical tests all 11 isolates were positive in the glucose fermentation test, six were positive in the lactose fermentation test and five were negative. Ten isolates were positive while one isolate was negative in the sucrose fermentation test. Six isolates were yellow slant and bud while three isolates were negative in the TSI fermentation test, one red slant with yellow bud, and one yellow slant with redbud. All of 11 isolates were positive in the catalase fermentation test (Table 4). According to the presumptive identification results, it was assumed that 11 isolates might belong to the genus “*Bacillus*” (Table 4).

**Table 4:** Biochemical tests for genus’s identification of bacterial isolates.

Bacterial isolates	Biochemical tests							
	Gram Staining	Motility Test	Citrate utilization test	Catalase	Glucose	Lactose	Sucrose	TSI
CD1	+	+	+	+	+gas	+	+	Both yellow
CD2	+	+	+	+	+	-	+ gas	Both yellow
CD3	+	+	+	+	+gas	+	-	-
CD5	+	+	+	+	+	-	+ gas	Both yellow
CD6	+	+	+	+	+	-	+ gas	Both yellow
ET1	+	+	+	+	+ gas	+ gas	+ gas	Both yellow gases produced
ET3	+	+	+	+	+	+	+	Both yellow
ET5	+	+	+	+	+ gas	+	+ gas	Slant red but yellow
IW2	+	+	+	+	+	-	+ gas	-
IW8	+	+	+	+	+	-	+ gas	-
IW9	+	+	+	+	+	-	+ gas	Slant yellow but red

CD = cow dung, ET = Effluent treatment, IW = Industrial waste.

### Optimization for cellulase production

**Fig 2:**
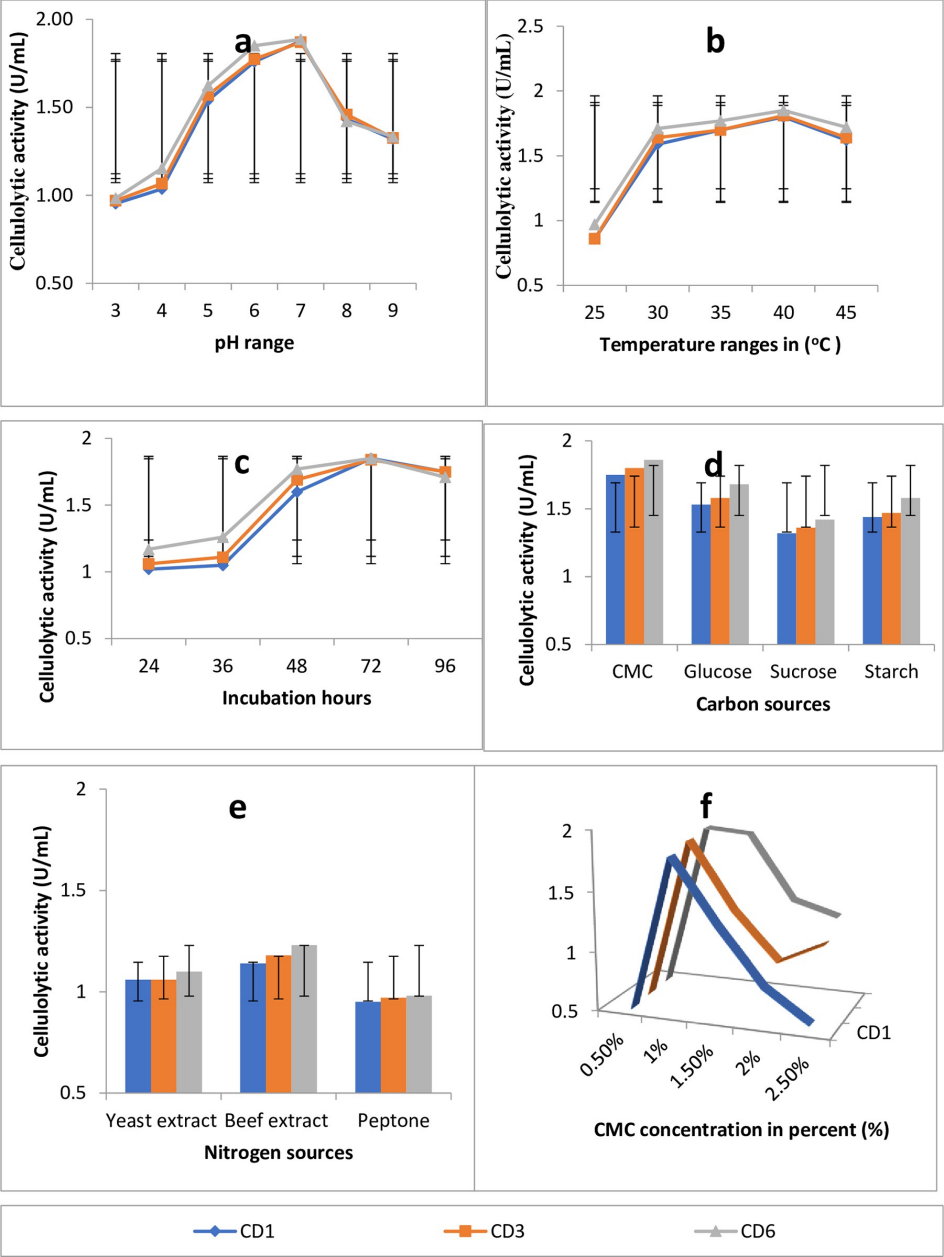
Optimization of the enzymatic activities of three cellulase-producing bacterial isolates on effects of PH range (a), temperature range (b), incubation time (c), carbon sources (d), Nitrogen sources (e), and CMC concentration(f). The values shown represent averages from triplicate experiments and the Cellulolytic activity of enzymes from different isolates showed a significant difference (*P*≤0.05) under different factors.

The effect of different factors on the activity of cellulose was determined by measuring cellulase efficiency at various temperatures, pH, incubation period, substrate concentration etc. Once the steps for identification of bacterial isolate at species level were done three isolates CD1, CD6 and CD3 which have produced the largest cellulolytic index (3.1, 2.9 and 2.87) respectively from the 11 cellulase-producing bacteria were selected for further study. Selected bacterial isolates (CD1, CD3 and CD6) were tested at various parameters of pH, temperature, incubation period, substrate concentration and carbon sources on cellulase production. In our study, described by Fig 2, cellulase-producing bacterial isolates showed maximum enzymatic activities at PH 6–7, temperature 35–45°C and incubation time of 48–72 hours and the lowest enzymatic activities were recorded at pH 3–4 and at pH>9, temperature 25–30°C and incubation time 24–36 hours. The highest cellulase activity was observed when CMC was used as a carbon source followed by glucose and starch and the lowest was observed when sucrose was used as a carbon source (Fig 2f).

## Discussion

The main technological hurdle to more widespread usage of cellulose resources is the lack of low-cost technologies for overcoming the recalcitrance of cellulosic biomass. A viable technique for overcoming this limitation involves producing cellulolytic enzymes and desired products in a single process step using a cellulolytic microbe or consortium [33]. This fact served as the primary impetus for this work, which aimed to separate and screen cellulolytic bacteria from various wastes and habitats. Using samples taken from waste from the Dashen brewery, forest soil, spent grain, industrial waste, and cow dung, a total of 34 unique colonies were isolated. 11 isolates out of 34 colonies were seen to provide a clear zone around their colonies. 11 isolates were identified utilizing a variety of morphological and biochemical testing. All of the isolates passed both gram staining and catalase fermentation tests and were positive. The isolates are most likely members of the genus *Bacilli* based on morphological and biochemical tests.

CD1, CD6, and CD3 which had the highest cellulolytic index were examined for their ability to produce cellulase under various conditions. The selected isolates were maximum at pH 6–7, and the least quantity of cellulase synthesis was seen at pH >9. These findings concur with those of [20] who indicated that the optimal pH was 7 and that pH 10 produced the least cellulases. This could be a result of the bacteria being neutrophilic. The highest amount of cellulase production was found at a temperature of 40°C, which was followed by a temperature of 35°C as the second-best temperature. On the other hand, the lowest amount of cellulase production was found at a temperature of 25°C. These findings support the findings of [10] who said that 40°C is the ideal temperature for cellulase synthesis. Depending on the studied microorganism, cellulase-producing bacteria have a wide range of optimum growth temperatures. The peak of cellulase synthesis was seen between 48 and 72 hours of incubation. 24 hours of incubation produced the least quantity of cellulase. These findings support the findings of [34], who claimed that the peak cellulase synthesis occurred between 48 and 72 hours. This shows that up to 24 hours of environmental adaption, no exponential growth, and incubation times greater than 72 hours revealed nutritional depletion.

The highest cellulase activity was observed when CMC was used as a carbon source and the lowest cellulase activity was observed when sucrose was used as a carbon source. These findings lined with [20] who said that CMC-supplemented medium saw the highest levels of enzyme synthesis [34]. The greatest level of cellulase production was seen when the beef extract was employed as a nitrogen source, followed by yeast extract and peptone. These findings conflict with those of [35] who claimed that yeast extract is the optimum nitrogen source for maximizing the synthesis of cellulase by different bacteria. 1%-1.5% CMC level had maximum enzyme activity, this result is in agreement with Farjana and Narayan’s (2019) in which the maximum cellulase activity was seen at 1% CMC concentration. 0.5% CMC concentration had low enzyme activity these findings disagree with those of [36], who claimed that Bacillus produces cellulase at a high level when CMC concentration is 0.5%. isolated bacteria from specific cellulosic sources have a high potential for producing cellulase enzymes, particularly with increased endo-β-1,4-glucanase activity. The identified isolates were Bacillus species through BLAST comparisons [37], which agrees with our current study.

## Conclusion

Nowadays, the number of brewery industries is increasing in Ethiopia and they generate a high amount of barley spent grain. This by-product is mainly used as low-value cattle feed or simply deposited as waste into landfills. In this study, cellulase-producing cellulolytic bacteria were isolated from forestry soil, cow dung, Dashen brewery waste, and industrial dump waste. 34 isolates were screened by staining their colonies using Congo red solution, and 11(32%) isolates were observed to give a zone of clearance around their colonies. About 23 (68%) of the isolates showed no cellulase activity. For secondary screening, three potential isolates were chosen based on the cellulolytic index. Among these isolates, Cow dung sample 1 (CD1) demonstrated the highest enzyme activity, producing a clearing zone of 4.5 mm and a height cellulolytic index of 3.1, and based on morphological and biochemical characteristics identified as *Bacillus sp*. Cellulase-producing bacterial strains can be grown under various optimized conditions. The optimal conditions for cellulolytic activity were a CMC concentration of 1%, a temperature of 40°C, an incubation period of 72 hours of, beef extract as a nitrogen source, and a pH of 7. Therefore, cow dung samples proved to be a good possible source of cellulase-producing bacteria. Increasing the number of isolates could also increase the chance of obtaining bacteria with interesting features and molecular characterization for bacterial isolates makes the study good.
